# Radiolabeling of Nanomaterials: Advantages and Challenges

**DOI:** 10.3389/ftox.2021.753316

**Published:** 2021-12-13

**Authors:** Wanqin Dai, Junzhe Zhang, Yun Wang, Chunlei Jiao, Zhuda Song, Yuhui Ma, Yayun Ding, Zhiyong Zhang, Xiao He

**Affiliations:** ^1^ CAS Key Lab for Biomedical Effects of Nanomaterials and Nanosafety, Chinese Academy of Sciences, Beijing, China; ^2^ CAS-HKU Joint Laboratory of Metallomics on Health and Environment, Institute of High Energy Physics, Chinese Academy of Sciences, Beijing, China; ^3^ School of Physical Sciences, University of the Chinese Academy of Sciences, Beijing, China; ^4^ Institute of Chinese Materia Medica, China Academy of Chinese Medical Sciences, Beijing, China; ^5^ Artemisinin Research Center, China Academy of Chinese Medical Sciences, Beijing, China

**Keywords:** radiolabeling, incorporation strategy, derivatization strategy, nanomaterials, radiotracer technique, *in vivo* stability

## Abstract

Quantifying the distribution of nanomaterials in complex samples is of great significance to the toxicological research of nanomaterials as well as their clinical applications. Radiotracer technology is a powerful tool for biological and environmental tracing of nanomaterials because it has the advantages of high sensitivity and high reliability, and can be matched with some spatially resolved technologies for non-invasive, real-time detection. However, the radiolabeling operation of nanomaterials is relatively complicated, and fundamental studies on how to optimize the experimental procedures for the best radiolabeling of nanomaterials are still needed. This minireview looks back into the methods of radiolabeling of nanomaterials in previous work, and highlights the superiority of the “last-step” labeling strategy. At the same time, the problems existing in the stability test of radiolabeling and the suggestions for further improvement are also addressed.

## Introduction

Nanotechnology has emerged rapidly during the past years in a broad range of product domains. It provides opportunities to manipulate or develop materials at nanoscale dimensions for a wide variety of applications (Liong et al., 2008; Chhowalla et al., 2013; Wu H. et al., 2020; Liu et al., 2021; Xuan et al., 2021), where conventional techniques may reach their limits. Nanomaterials (NMs) are inevitably being released into the environment during the processes of production, transport, use, disposal and recycling, and subsequently into human bodies. In addition, human may be unintentionally exposed to some natural or incidental NMs (Hochella et al., 2019; Wang H. et al., 2020), and sometimes even intentionally, to some medical NMs (Martins et al., 2020). Regardless of the sources and exposure routes of NMs, tracing and quantifying the biodistribution of NMs is fundamentally important to wide-ranging fields from nanotoxicology to drug delivery (Wang et al., 2013).

In general, the methods for NMs measurement in biological samples depend on the chemical composition and peculiarities of the structure of NMs, and there is no universal technique to date suitable for all NMs. Among those techniques developed for NMs quantification and imaging (as shown in Table 1), isotope tracing, especially radioactive tracing, is one of the most powerful tools available for assigning a source and tracking their distribution from nano to global scale. In addition, radiolabeled NMs have drawn considerable attention in the fields of nuclear medicine and molecular imaging, drug delivery, and radiation therapy (Ge et al., 2020; Wu S. et al., 2020). Whether in a radiotracing study or construction of a radioactive theranostic nano-plat, radiolabeling of NMs is always a prerequisite and a major challenge. Therefore, researchers should weigh the necessity of using radioactive tracing, how to choose nuclides, what kind of connection strategy to adopt, and how to minimize the inherent defects of radiotracing methods.

**TABLE 1:** Techniques for NMs quantification or imaging.

Technique	References(s)
optical/electron microscopic imaging	Lee et al. (2011); Tsoi et al. (2016); Guillard et al. (2020); Sun et al. (2020)
photoacoustic imaging	Bouchard et al. (2009); Kim et al. (2011)
Raman spectroscopy	Zavaleta et al. (2009); Nicolson et al. (2019)
optical emission spectrometry	Kückelhaus et al. (2005)
mass spectrometry	Cai et al. (2016); Meermann and Nischwitz (2018)
MR^a^ imaging	Sun et al. (2008); Lee et al. (2011)
Ferromagnetic resonance	Lacava et al. (2001); Levy et al. (2011)
neutron activation analysis	Zinicovscaia et al. (2018)
isotope tracing	He et al. (2010); Zhang et al. (2011); Chen et al. (2017); Zhang et al. (2019)
PET/SPECT^b^	Tseng et al. (2014); Gao et al. (2015); Ni et al. (2018)
synchrotron radiation XRF^c^ or STXM^d^	Qu et al. (2011); Zhang et al. (2012); He et al. (2013)

Note: ^a^magnetic resonance.

b positron emission tomography / single-photon emission computed tomography.

c X-ray fluorescence spectroscopy.

d scanning transmission X-ray microscopy.

## Advantages and Disadvantages of Radiotracer Technique

Isotope tracing, especially radioactive tracing, shows superiority over the other techniques developed for NMs quantification and imaging due to its following features (for more focused discussion, only radiotracer technique will be mentioned below):

High sensitivity. Radiotracer technique has a much greater sensitivity compared to conventional fluorescence labeling (Yin et al., 2017), and can be associated with a range of detection methods, including *γ* spectrometry, scintillation counting, PET and SPECT. For example, radioactive ^141^Ce labeling and HPGE *γ* spectrometry were used to quantify the distribution of ceria nanoparticles (NPs) in rat after intratracheal injection, and a detection limit better than 1 pg/g was achieved (He et al., 2010).

Great accuracy and reliability. The accuracy and reliability of radiotracer technique relies on the ultra-low background noise in radioactivity detection, therefore, the specific signals of the labeled NMs could be easily distinguished from the interference or artifacts from natural or background-level components (Zhang et al., 2009). Without labeling, the signals of carbon NMs would be completely masked by the organic or inorganic carbon in environmental and biological matrices (Chen et al., 2017). The detection of some metal-based NMs is also limited by the naturally high background. For example, the quantification of iron oxide NPs *in vivo* might be interfered with by endogenous iron (Patil et al., 2015).

Large penetration depth. Since the decay of the majority of radioactive isotopes involves the emission of high-penetrating *γ*-ray, radiolabeled NMs in bulk samples could be directly detected without pre-cutting, crushing or digesting the samples. This greatly reduced the difficulty of tracing and quantifying NMs in complex matrices. When matched with PET or SPECT, radiotracer technique could even provide a non-invasive, whole-body, real-time, and dynamic imaging capability (Yu et al., 2017; Abdollah et al., 2018). It makes possible to quantitatively measure the NMs concentration in various organs over time, which provides invaluable information for NMs pharmacokinetics (also known as particokinetics) or NMs-based diagnosis and therapy.

However, radiotracer technique has some inherent disadvantages. Often, the selection of radiolabeling methodology will be limited by radionuclide/equipment availability, as this type of work requires expensive facilities and strict qualifications (Pellico et al., 2021). After radioactive contamination, matrices and animals must be maintained or disposed in a way that satisfies safety requirements for human staffing. But the greatest difficulty of radiotracer technique lies in the radiolabeling of NMs, because the experimental operation can be time-consuming and laborious, and requires radiation shielding for health and safety considerations.

## Radiolabeling Strategies

An ideal radiolabeling strategy should be easy, fast, robust, and highly efficient and must make only minimal changes to the original properties of NMs. Radiolabeling strategies could be classified according to the type of NMs, the radionuclide and/or the final application. In this review, labeling strategies are divided into two categories according to the timing of adding radionuclides: the incorporation strategy and the derivatization strategy. It needs to be clarified in advance that this simple classification is only for the convenience of discussion, and some exceptions will also be mentioned case by case.

The incorporation strategy incorporates the radionuclide into the structure or the core of NMs *via* radiochemical synthesis, using a synthetic route exactly the same as the NMs to be labeled, only partially replacing one of the cold precursors with a hot nuclide. For example, Zhang et al. (2011) radiolabeled ceria NPs by radiochemical synthesis of ^141^CeO_2_ from a mixture of ^140^Ce(NO_3_)_3_ and ^141^Ce(NO_3_)_3_, and Pellico et al. (2016) radiolabeled iron oxide NPs (IONPs) by doping ^68^Ga in the core of IONPs. The as-synthesized radioactive NMs have almost, if not totally, the same physicochemical properties as the unlabeled NMs. Therefore, incorporation strategy is also called intrinsic radiolabeling method in some previous reports (Goel et al., 2014; Chakravarty et al., 2018; Fach et al., 2021), which could essentially guarantee an accurate reflection of NMs behavior by detecting the radioactive signals. In addition to radiochemical synthesis starting from the cold-hot precursors, radionuclide can be incorporated into the NMs through surface elemental exchange, or radionuclide deposition in cage-like/mesoporous NMs (Goel et al., 2014; Tang et al., 2019; Korany et al., 2020). NMs can also be radioactively activated *via* thermal and epithermal neutron bombardment before the tracing study (Antsiferova et al., 2015). However, neutron activation may bring unexpected thermal denaturation (especially to the organic functional groups on the particle surface) and structural damages (Yin et al., 2017), therefore will not be discussed in this review.

Another category of radiolabeling uses derivatization strategy, in which non-radioactive NMs are synthesized first, and then radionuclide is conjugated to the surface of the pristine NMs by physical (e.g., adsorption) or chemical (typically *via* a radionuclide chelator anchored on the NMs surface) means. For example, MoS_2_ NPs were radiolabeled with ^64^Cu with the help of bifunctional p-SCN-Bn-NOTA (Dong et al., 2018), and IONPs were radiolabeled with ^69^Ge *via* surface adsorption (Chakravarty et al., 2014). This strategy is versatile and can incorporate various radionuclides of choice onto the surface of NMs. However, the derivatization of NMs could potentially change the surface properties of the NMs to be radiolabeled and subsequently alter their environmental or biological behaviors. In other words, the research should always keep in mind that he/she is tracing the NMs with radionuclide or radionuclide-chelator complex on their surface, not their pristine form. (Das et al., 2021).

But then again, surface-unmodified pristine NMs have very limited biomedical applications, while NMs with multiple surface-functionalized have been extensively explored to achieve better dispersibility, targeting and versatility. If considering the chelator for radionuclide as an intrinsic component of the NMs to be radiolabeled, the attachment of radionuclides to NMs could also be regarded as an intrinsic radiolabeling. With this premise, the derivatization strategy could highlight its advantages over the incorporation strategy in introducing radionuclide into the NMs to be labeled as late as possible in the sequence. It allows researchers to synthesize the pristine NMs non-radioactively, modify the surface and multi-functionalize the NMs non-radioactively, and then conjugate radionuclide to the NMs in the last step. Therefore, the derivatization strategy can be optimized as the “last-step radiolabeling”. In this way, the experimental procedures that require radiation shielding are minimalized, and the specific activity required for radiolabeling is also minimalized due to a shortened interval between the radiolabeling and the tracing study (Zhang et al., 2019). In addition, the derivatization strategy can create considerably more leeway in choosing radionuclides and experimental procedures for radiolabeling. These features make derivatization strategy relatively more attractive than incorporation strategy due to the flexibility in design, ease of operation, low radiation risk, low cost and time-saving.

## *In Vivo* Stability of Radiolabeling

Despite the above-mentioned advantages of the derivatization strategy, there is still a major concern about the potential detachment of radioisotopes *in vivo*, which could lead to problems such as off-targeting and false positives. After all, the derivatization strategy binds radionuclides to the surface of the NMs, where radionuclides would directly interact with bio-milieu. Generally, organic radiotags linked by strong covalent bonds, like ^11^C-methylations, ^18^F-based prosthetic groups, and ^14^C-taurine (Deng et al., 2007; Lamb and Holland, 2018; Pellico et al., 2021), would not be easily dissociated from the NMs *in vivo*. But this is not the case for those radiometals. The large family of radiometals provides the greatest diversity in the selection of radiotags, but also poses the biggest challenge in radiochemical stability because radiometals in their ionic forms might be released from NMs surface *in vivo*. Therefore, the last part of this minireview will discuss the possible *in vivo* detachment of radiometals from the surface of NMs.

In the existing literature, the radiochemical stability of the NMs-radiometals complex has always been investigated *ex vivo* as follows: after the direct (chelator-free) or indirect (typically chelator-based) chemical bond formation between the radiometals and the NMs surface, the complex is incubated in simulated body fluids (i.e., serum or PBS at 37°C) for several hours or overnight, and then the detached radiometals are collected and quantified by means of centrifugation, chromatography, or liquid chromatography (Korany et al., 2020; Wang L. Z. et al., 2020; Papadopoulou et al., 2021). However, one of our work just demonstrated that some features of the particokinetics may lead to a previously underappreciated loss in the *in vivo* stability of radiolabeling (Zhang et al., 2019). Once the radiolabeled NMs enter the bloodstream, they are most likely to be quickly removed by mononuclear phagocyte system (MPS), and trapped in the endosomes of MPS cells. These endosomes would develop into lysosomes, with a drop of luminal pH from 7.4 to 6.5 within a few minutes, and 6.4 to 4.0 within 1 h (Wang et al., 2017). The rapid and large drop of the local pH may cause significantly more radiometals to detach from the surface of NMs than the case in neutral solutions. The effects of low pH will be discussed separately for specific radiolabeling methods.

**FIGURE 1:**
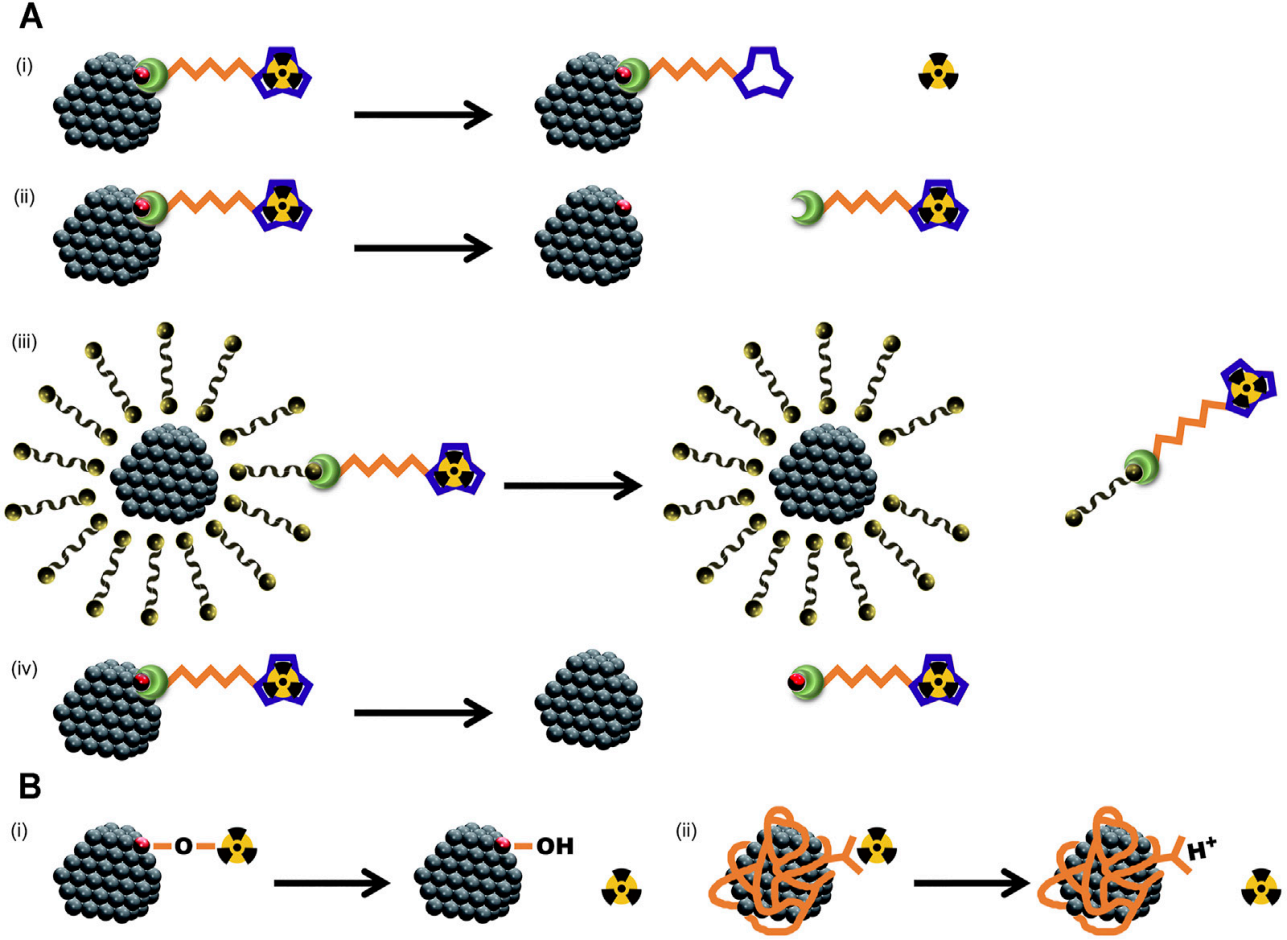
Schematic diagrams for different types of radiometal detachment from NMs *in vivo*, especially at low pH milieu. **(A)**, reasons for the detachment after chelator-based radiolabeling: **(i)** compromised chelation; **(ii)** failed conjugation; **(iii)** weak bond between NMs and radiometal; **(iv)** decomposition of the NMs. **(B)**, reasons for the detachment after chemical adsorption radiolabeling: **(i)** desorption due to proton competition; **(ii)** carboxyl group protonation.

Classic methods for labeling NMs with radiometals rely on the functionalization of NMs surface with traditional metal ion chelators. Radiochemists have long been committed to synthesizing and improving bifunctional chelating agents, with a chelator (like polyaminocarboxylate groups) at one end and a chemically-reactive linkage group at the other end (Pellico et al., 2021). When the chelator matches the radiometals, the radiolabeling could stay stable during the *ex vivo* measurement of radiochemical stability. However, inappropriate choice in linkage and the local low pH may lead to the detachment of radiometals from NMs in the following cases:

1) The protonation of chelator’s carboxyl and amine groups may compromise the *in vivo* stability of the chelation (Figures 1A, i. (Liu and Edwards, 2001; Ramli et al., 2009).
2) The newly formed bond between the linkage group of bifunctional chelating agent and the NMs is too weak to maintain stability *in vivo* (like some ether bonds or ester bonds, Figure 1Aii).
3) When the chelators are not linked to the surface atoms of NMs, but to the small molecules that constitute the surface coating of NMs through hydrogen bonding or amphiphilic self-assembly, then the chelator-radiometal complexes may detach from the surface of NMs together with the coating molecules (Figure 1Aiii).
4) The chelators are directly linked to the surface atoms of NMs, but the superficial layer of NMs undergo a decomposition *in vivo* (especially when in a low pH milieu, Figure 1Aiv).

Besides chelator-based radiolabeling, surface chemical adsorption of radiometals is simple and easy to implement, and has received great attention as a chelator-free radiolabeling method. In the existing examples (Chakravarty et al., 2014; Lamb and Holland, 2018), the NMs to be radiolabeled often have a surface coating (like PAA), and therefore, it is difficult for us to distinguish whether the radiometals are directly adsorbed on the surface of metal oxide NPs (forming M-O-M bond) or combined with the carboxyl groups in the surface coating. Nevertheless, the protonation of either the particle surface or the carboxyl groups in the low pH could lead to the detachment of radiometals (Figures 1Bi,ii). When the PAA coating is loose, or the NMs are easily decomposed *in vivo*, the chemically adsorbed radiometals may also undergo a detachment in the manners similar to those shown in Figures 1Aiii,iv.

Based on the above discussion on the causes of radiometal detachment *in vivo*, we here propose several improvements: 1) We should continue to improve bifunctional chelating agents with pH-insensitive stabilities of both the chelation and the linkage. 2) If we want to radiolabel degradable NMs *via* surface derivatization but do not want to hinder the degradation of the NMs, we could encapsulate the NMs with a mesoporous shell or long organic chains with a high degree of cross-linking, and then connect the radionuclides and the NMs coating through strong and pH-insensitive bonds. 3) Only when the NMs to be radiolabeled could remain undegraded *in vivo* for a long time, can we directly connect the radiometals to the surface atoms of NMs through strong and pH-insensitive bonds. 4) The *ex vivo* measurement of the radiolabeling stability should be conducted not only in the simulated neutral body fluids, but also in the artificial lysosomal fluid. In short, we must pay attention to the differences in metabolism between nanomaterials and molecules, as well as the resulting different requirements for radiolabeling. Furthermore, the introduction of a higher level of automations and even artificial intelligence in the design and practice of radiolabeling will definitely bring new options for the radiotracing study of NMs (Zhang et al., 2019; Xu et al., 2021).

To sum up, this minireview looks back into the methods for NMs radiolabeling, and highlights the superiority of the “last-step radiolabeling” *via* derivatization strategy. Meanwhile, we emphasize here a pH-related detachment of radionuclides from the NMs that has been underappreciated previously. Since the radiolabeled NMs will encounter a low pH milieu soon after exposure, radiochemical stability in acidic environment is essential for radiolabeling. This minireview also calls for a careful re-examination of the previous radiotracing results, as well as further optimization on the radiolabeling methods for NMs.
